# Patient Compliance during 24-Hour Dual pH Probe Monitoring for Extraesophageal Reflux

**DOI:** 10.5402/2011/703936

**Published:** 2011-12-11

**Authors:** Joy Musser, Lisa Kelchner, Jean Neils-Strunjas, Marshall Montrose

**Affiliations:** ^1^Department of Communication Sciences and Disorders, University of Cincinnati, P.O. Box 670394, Cincinnati, OH 45267, USA; ^2^Department of Molecular and Cell Physiology, University of Cincinnati, P.O. Box 670576, Cincinnati, OH 45267, USA

## Abstract

*Objective*. During ambulatory 24-hour dual pH probe monitoring for suspected extraesophageal reflux (EER), patients are responsible for indicating relevant study events. Study interpretation relies on patient accuracy and compliance to test instructions. This study sought to explore patient compliance during pH probe monitoring and evaluated the utility of a Post-Evaluation Questionnaire as a clinical tool. *Participants and Methods*. Participants were prospectively studied during 24-hour dual pH probe monitoring. Participants used both a food diary and monitor settings to indicate relevant study events. Following pH testing, participants completed a Post-Evaluation Questionnaire regarding test experiences. *Results*. Eighty-two participants completed the study. Means and standard deviations were calculated for individual responses on the Post-Evaluation Questionnaire. Means indicate high participant accuracy for study events, and adherence to typical activities and diet over the testing period. Factor analysis was performed on the Post-Evaluation Questionnaire items and identified two factors: “typical experiences” and “times forgot.” Cronbach's alpha demonstrated acceptable reliability levels for questions related to “typical experiences,” but poor reliability for “times forgot” questions. *Conclusions*. Assessment of participant compliance during pH probe testing can quickly and easily be completed through a Post-Evaluation Questionnaire. Participant compliance can be assessed for improved study interpretation.

## 1. Introduction

Extraesophageal reflux (EER) is the backflow of stomach contents into the larynx, pharynx, or oral cavity. This type of reflux has been implicated in complaints such as hoarseness, chronic cough or throat clearing, a “lump in the throat” sensation, and difficulty swallowing [1] as well as laryngeal findings of contact ulcers and granulomas, laryngeal carcinoma, and subglottic stenosis [2].

Despite other available and seemingly superior technologies such as impedance testing, the current “gold standard” for EER diagnosis continues to be 24-hour dual pH probe monitoring. This procedure involves placing a catheter through the nose and into the esophagus. Two sensors, proximally and distally located within the catheter, detect the pH level or acidity in the distal esophagus and hypopharynx. EER is diagnosed when stomach contents are shown to flow upwards from the stomach to the distal esophagus, and subsequently to the proximal esophagus and into the hypopharynx. Measurements of pH are recorded to a small, portable device that is worn on a belt during the study. Data obtained from the study include the frequency, duration, and acidity levels of distal-proximal acid reflux events. It is well accepted that the diagnosis of EER can be elusive given its nonspecific symptoms and poor agreement on physical findings [2–4]. Furthermore, recent reports have illuminated the discordance between EER symptoms, physical findings, and pH probe results [5–8].

During the 24-hour pH probe study, patient compliance to test instructions is a critical component of test accuracy. Patients must utilize several key settings on the pH monitor device. These settings indicate ingestions, body position (upright, supine), and the occurrence of EER symptoms (hoarseness, cough, throat clear, etc.) occur. Patients are instructed to select the ingestion setting *as soon as* eating/drinking begins and leave this option selected for the duration of the meal. Patients must indicate “ingestion” *every time* food or liquid (excluding water) is swallowed. If ingestion is not occurring, the default “no ingestion” setting is selected. These events are recorded by the pH monitor and uploaded with study data. When reviewing the pH tracing, acid pH spikes below pH 4 observed at the proximal and distal sensors during these self-reported ingestions are ignored; conversely, acid pH spikes demonstrating distal-proximal directionality below pH 4 that fall *outside* of ingestion periods are regarded as true EER events. If patients are inaccurate in reporting the time and duration of ingestions, study results may be reported as falsely negative or falsely positive. 

Patients are also encouraged to maintain daily routines involving types of food, meal times, and typical activities in order to obtain a representative sample during the 24-hour period. It is believed that the standardization of meals or imposed dietary regimens may significantly disrupt the typical pattern of acid reflux and symptoms in patients [9, 10]. Lim et al. [11] administered a questionnaire to patients with suspected gastroesophageal reflux following the completion of a 24-hour pH probe study, in which areas of food, activity, and distress were analyzed. No significant differences were found between patients with positive or negative pH studies in response to these areas, indicating little to no effect of daily routines on study results. Adherence to test instructions was not assessed. This type of questionnaire has not been used prospectively with patients with *EER* and may provide useful information as the pH study is interpreted. To determine if information at the time of testing is representative, the degree to which patients diverge from daily routines during pH testing needs further research.

### 1.1. pH Monitoring Analysis

Software packages generally provide a standard readout of pH levels at the distal and proximal sensors, such that the tracings for each can be reviewed simultaneously (see Medtronic, Minneapolis, Minn, USA; Sandhill Scientific, Highlands Ranch, Colo, USA). Time is displayed along the *x*-axis over the course of the study period and the reviewer has the ability to examine the tracings across various time increments, and as often as every 30 seconds. The pH levels ranging from 0 to 8 for both the distal and proximal sensors are represented along the *y*-axis. Relevant study events such as body position, the occurrence of ingestions, and symptoms can also be represented. These features allow the reviewer to assess the patient's individual pattern of reflux.

Software protocol allows the reviewer to easily exclude meal periods, so that analysis is not confounded by a multitude of false-positive pharyngeal reflux events occurring during ingestion. In these instances, a pH drop will first be seen at the proximal probe followed a few seconds later by a pH drop at the distal probe, demonstrating proximal-distal directionality. Furthermore, a “true” pharyngeal acid event is one that the start of the esophageal event occurs *simultaneously with* or up to 20 seconds *before* the pharyngeal event, demonstrating distal-proximal directionality [10, 12]. In addition to the artifacts imposed by meal periods, occasionally isolated proximal pH drops (in absence of a concomitant esophageal pH drops) can be seen and are referred to as “psuedoreflux” and originate from the monitoring circuit itself [10]. Given the possibility of these study artifacts, many authors stress the importance of manual/visual review of each study tracing [10, 13]. Recently, Harrell et al. [14] investigated the impact of artifacts in hypopharyngeal pH monitoring. They found that as many as 80% of pH drops at the proximal sensor were potential artifacts such as out of range pH, pH drift, or an isolated proximal pH drop. After making these exclusions, the diagnostic yield of pH monitoring in the hypopharynx dropped to less than 50%. Subsequent to their tedious review, it would appear as if all artifacts could be accounted for and eliminated from analysis thereby improving pH monitoring specificity. However, researchers have yet to address the patient's compliance and accuracy during testing. Given that standard procedure does not control for diet during the 24-hour period, even the most careful review still *assumes* that the patient is accurate and precise in reporting ingestion, both through button presses on the monitor device itself and in food diary entries. Accounting for the patient's role during testing with regard to false-negative or false-positive pharyngeal acid reflux events has yet to be investigated.

### 1.2. Purpose of the Study

Patient compliance with test procedures is necessary in order for pH probe testing to be interpreted with a certain degree of confidence. Patient compliance would seem to be a considerable factor in the reliability and validity of pH probe testing as the “gold standard” for EER diagnosis and should not be based on clinician assumptions. Given the poor agreement between clinical tools used in the diagnosis of EER, patient compliance during pH monitoring is an area warranting investigation. *Patient noncompliance may lead to inaccurate interpretation of study results leading to a false or a missed diagnosis of EER, and resulting in inappropriate treatment recommendations.* Although the importance of accurate patient reporting is implied in test instructions, it is unknown how precise patients are with regard to indicating start/stop times of all liquid and food ingestions, both on the monitor device itself, or within their meal diary. Surprisingly, no single research study has investigated this issue. 

The purpose of this study was to explore how participants self-rated their compliance and accuracy with pH probe test procedures, and adherence to typical routines using a postevaluation questionnaire and patient interview, in the immediate posttest period. In addition, the questionnaire itself was assessed for its use as a clinical instrument.

## 2. Materials and Methods

### 2.1. Participant Selection

The protocol for this study was approved by the Institutional Review Board of the University of Cincinnati and informed consent was obtained from all participants. Participants were enrolled from a private Midwest otolaryngology practice. Participants were referred for pH evaluation by their otolaryngologist based on symptoms suggestive of EER (hoarseness, chronic cough or throat clearing, a “lump in the throat” sensation, and difficulty swallowing) and subsequently were eligible for enrollment in this study. All patients who were referred for 24-hour dual pH probe evaluation by an otolaryngologist, regardless of their potential enrollment in the study, were screened for participation.

### 2.2. Diagnostic Testing Protocol

In order to ensure consistency, all study instructions and related procedures were accomplished with a standard protocol and performed by the principal investigator in the same procedure room within the participating otolaryngology practice for each enrolled participant. The proximal sensor was placed under visual guidance using the Smit technique, which has been consistently noted to place the upper probe at the location of the upper esophageal sphincter [15]. Following pH catheter insertion, all participants received standard verbal instructions to maintain their typical daily routines and eating habits, and to change settings on the monitor device to indicate ingestions, symptoms, and body position (upright or supine). Participants' diet over the study period was not manipulated in order to capture a representative sample. Participants kept a food diary, including the beginning and end times of ingestion, during the 24-hour testing period (Appendix A). In addition, they also reported whether they consumed foods or liquids that they believe aggravate their individual symptoms of acid reflux. The importance of accurately recording start/stop times of ingestion, both on the monitor device and on the food diary, was emphasized to the participant, as with standard test instructions. Instructions were provided in verbal and written forms.

Directly following catheter removal, participants completed a brief, easily administered (5.8 Flesch-Kincaid grade level) questionnaire about the 24-hour pH probe study experience (Appendix B). Questionnaire items were developed based upon clinical queries generated from pH probe testing and represent the typical information that is requested from the patient, as is standard patient care within the participating practice. Participants were encouraged to respond honestly and without fear of penalty, in order to provide their physician with the necessary information to determine the presence/absence of EER on pH study. This questionnaire was completed prior to any manual review by the primary investigator, so as not to influence the participants' responses regarding accuracy or compliance. The principal investigator then reviewed the participants' food diary and ingestion start/stop times with the participant. When possible, missed or delayed ingestions were then corrected by manually entering the times into the data software. For example, if the food diary indicated that the participant finished eating at 7:00 PM but this was not recorded by a setting selection within the data, the investigator would regard this as a missed button press. Because the participant had recorded the meal ending time on the food diary, the investigator would regard this as a discrepancy (not inaccuracy) and could then manually supply this information into the program for analysis.

### 2.3. Statistical Analysis

Descriptive statistics will be calculated for all Post-Evaluation Questionnaire (Appendix B) responses, and factor analysis will be performed. Finally, Cronbach's alpha reliability will be conducted. All statistical analyses will be calculated using IBM SPSS Statistics (Chicago, Ill, USA).

## 3. Results

The experimental group participants included 22 (31%) males and 50 (69%) females ranging in age from 27 to 90, with a mean age of 57.72 (SD = 14.56, median of 56.5). Ten asymptomatic volunteers also participated in this study and included 3 (30%) males and 7 (70%) females ranging in age from 22 to 58, with a mean age of 39.9 (SD = 10.69, median of 39.5). Asymptomatic volunteers were enrolled as part of a larger study [5], and their data were included for the purposes of the current study. Volunteers were in good general health, had no history of current treatment by an otolaryngologist, had not noted heartburn or acid regurgitation more than three times per month, had no routine use of antireflux medication, had a negative history of smoking at least for the past five years, and were not pregnant or lactating. Only those volunteers who had a total Reflux Symptom Index (RSI) [16] score less than or equal to 13 were enrolled into the control group. In total, eighty-two participants completed this study.

Means and standard deviations were calculated for individual responses on the Post-Evaluation Questionnaire (Appendix B) for questions 1–7. Means reflect 0 to 5 Likert scale responses. Results are shown in Table 1. A mean of 2.11 for question number 1, “Overall level of discomfort” (where 0 = No discomfort, 5 = Severe discomfort) indicates that overall, participants experienced mild discomfort over the testing period. Means for questions 2, 3, and 7, “Typical daily activities,” “Typical diet,” and “Typical symptoms over past 24-hours,” respectively, (where 0 = very typical, 5 = very atypical/very different) indicate that overall, participants adhered to typical activities and diet and experienced typical symptoms over the testing period. Means for questions 4, 5, and 6, “Forgot to change setting for body position,” “Forgot to change setting for eating/drinking,” and “Forgot to use symptom button,” respectively, (where 0 = never, 5 = nine or more times) indicate that participants almost never forgot to indicate these events. Question  8 was excluded from analysis due to a large percentage (67%) of missing data.

**Figure 1:**
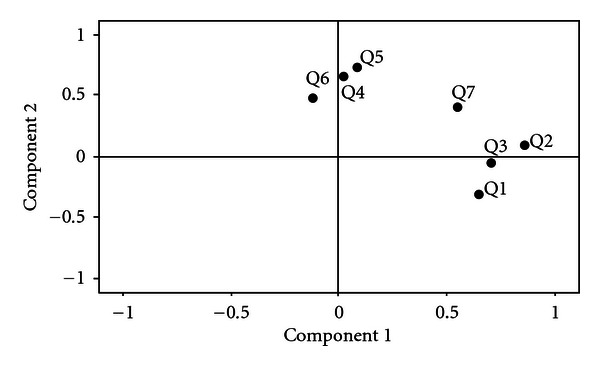
A factor analysis of questions  1–7 of the Post-Evaluation Questionnaire. Q1: Discomfort level; Q2: Typical activities; Q3: Typical diet; Q4: Forgot body position setting; Q5: Forgot eating/drinking setting; Q6: Forgot symptom button; Q7: Typical symptoms.

**Figure 2:**
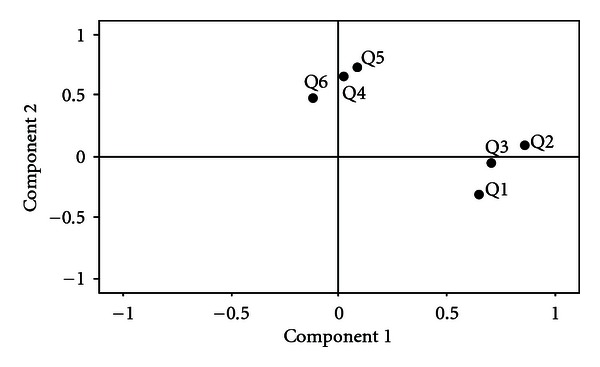
A factor analysis of questions  1–6 of the Post-Evaluation Questionnaire. Q1: Discomfort level; Q2: Typical activities; Q3: Typical diet; Q4: Forgot body position setting; Q5: Forgot eating/drinking setting; Q6: Forgot symptom button.

In order to uncover the latent structure of the Post-Evaluation Questionnaire, a factor analysis of the seven items was performed. Factor analysis can be used to validate a scale or index by demonstrating that constituent items load on the same factor. Direct oblimin rotation produces a simple structure of the factor loading matrix and makes the results easier to interpret. Results are presented in Figure 1. Direct oblimin rotation revealed two uncorrelated, underlying factors: “typical experiences” and “times forgot.” Questions  1–3 represent a “Typical Experiences” factor. Question  1 relating to participant discomfort during pH testing was included as a “typical experience” because the level of comfort during testing is a consideration when assessing the typicality of a participant's day. Questions  4–6 represent a “Times Forgot” factor. Results indicated that this questionnaire simultaneously evaluated these two different concepts. Question  7 did not correspond to either factor well, as seen in the plot below. Question  7 was subsequently eliminated from the factor analysis and results are displayed in Figure 2. Cronbach's alpha reliability was conducted to evaluate the internal consistency of the above factors. Cronbach's alpha reliability of the three questions item “Typical Experiences” factor is 0.643 and regarded as “acceptable” for an exploratory study [17]. Cronbach's alpha reliability of the three questions item “Times Forgot” factor is 0.313, demonstrating poor internal consistency. Results indicate the Post-Evaluation Questionnaire items related to “typical experiences” (Q1–3) are adequately measuring the same underlying construct, whereas questions related to “times forgot” (Q4–6) do not.

## 4. Discussion

The primary aim of this study was to explore patient behavior, namely, compliance and accuracy, during 24-hour pH probe monitoring using a posttest questionnaire and clinician interview. The internal consistency of the Post-Evaluation Questionnaire as a potential clinical tool to determine issues of patient compliance was also evaluated. 

Patient compliance has become an important issue in healthcare [18]. Efforts to assess patient compliance is critical in medical research since adherence to study protocols can have profound effects on results [19]. One study that investigated compliance with oral antipsychotic medication regimens found a weak correlation between subjective measures, such as physician or patient self-report, and objective data [20]. Another study involving memory training interventions examined the factors that influenced training outcomes. Their findings show that increased compliance was predicted by health (higher vitality, fewer functional limitations), education (advanced degrees), and self-efficacy (higher self-efficacy) [21]. Jerant et al. [19] found that psychological factors very likely affect research data. Specifically, participants with higher levels of “agreeableness” and “conscientiousness” tended to be more compliant. 

During a 24-hour pH probe study, patients are commonly asked to keep food diaries in order to record ingestions throughout the testing period. These diaries provide an additional way to report a patient's intake beyond what has been recorded using the ingestion button on the pH monitor device. However, study interpretation relies upon accurate reporting of ingestions during the study. In past studies examining acid reflux on pH monitoring, test protocol includes instructions to the patient such as “continue your typical diet,” “use the [pH] recorder's meal indicator when eating or drinking,” and “record meals in a diary” [9, 10, 14, 22]. One study that did not utilize a diary, reported that the defined meal stop/start times were “stated by the patients” [12], which would require precise recall. It is unknown how any inaccuracies or discrepancies were resolved. In these examples, investigators have seemingly *assumed* that participant's recall or food diaries reflect full participant compliance and precision when assessing study data for evidence of EER. 

In the current study, 82 participants completed a short questionnaire (following the removal of the pH catheter) rating their 24-hour test experiences and compliance with testing instructions. This study design was strengthened by its use of one examiner for all testing procedures for consistency of test instructions. Furthermore, each participant's food diary was reviewed with the sole examiner during manual data review of the study to ensure that all written and button-reported ingestions could be time-matched, and discrepancies were systematically resolved. Common discrepancies included missing button presses indicating the stop/start of an ingestion as determined by the food diary and/or participant report, and mildly delayed (up to 5 minutes) reporting of an ingestion. Admittedly, the potential for recall bias is present when data rely upon patient recollection. Despite this, the overwhelming majority (90%) of participants were cognizant of these minor discrepancies and were forthcoming and thorough with the corrections when reporting them to the primary investigator. In the few cases of wide ingestion-related discrepancies (missing stop/start times, long reporting delays), the primary investigator noted this within the data for special consideration during manual review of the data and study interpretation. In most of these instances, the presence/absence of EER could still be determined based on the remaining data. 

Some reports have cited an abandon of typical routines during pH probe testing [23, 24], but the current study's results for the “typical experiences” questions (Q1–3) reflected that despite mild discomfort, participants' typical routines were not significantly altered by the presence of the pH catheter. In fact, many participants returned to the workplace with the pH catheter in place. One female participant even hosted her monthly dinner party, while another proceeded with her daily aerobic exercise. They also reported experiencing typical EER symptoms (Q7), which gives credence that the testing period “caught” their symptoms and possible EER events. One male participant likened this to taking his car into the shop to have a troublesome “clunk” noise checked out; he was dually relieved that the “clunk” was also heard by his mechanic and that his “burning throat” was caught on tape, so to speak. This is supported by a study in which test day symptoms in suspected GERD patients undergoing pH probe monitoring were assessed. Symptoms that were “typical” or “worse than typical” were more likely to produce abnormal pH findings, while normal pH probe test results were found in patients who reported symptoms that were “better than typical” [25]. This finding provides additional support for the inclusion of patient ratings of their test period experiences to assist with the interpretation of pH monitoring results. Perhaps for those pH studies that are negative, but for whom patients self-rate “better than typical” symptoms, a repeat study could be performed. In addition, participants reported mild-moderate discomfort (Q1), although many reported to the examiner that the presence of the probe was more of a “nuisance” than actually painful. Furthermore, this group of questions demonstrated an acceptable level of internal consistency reliability, showing its promise as a useful clinical tool. Based on these findings, it would seem that the 24-hour testing period is a representative one in which to evaluate EER, and these postevaluation questions successfully reflect this finding. 

As a group, results for the “times forgot” questions (Q4–6) demonstrated high self-reported compliance to monitor and food diary use for relevant study events. Of significance with regard to study interpretation, were findings for question number 5 (“How many times did you accidentally forget to change the setting for an eating/drinking event over the past 24-hours?”). Results show a mean score of 0.55 (0.57 SD), indicating that participants “never forgot,” or “forgot once” to accurately report ingestions. Admittedly, asking participants to recall how often they forgot (to change a monitor setting) is difficult, but this finding has direct bearing on how clinicians interpret pH spikes within the data since reflux events below a pH 4 during a reported meal period are routinely omitted from analysis [26]. For example, a patient completes a meal at 7:00 PM but fails to indicate this on the pH monitor until 7:30 PM. If EER were to occur directly following the meal, this would be regarded as normal pH fluctuations due to ingestions and would therefore be a missed EER event. 

Unfortunately, there is generally poor agreement between existing clinical tools used to diagnose EER, such as the reliability of rating physical findings and 24-hour pH probe monitoring [5–8]. The compliance-type questions used in this study were developed as the first phase of a posttest instrument. Construction of an instrument to assess patient compliance is motivated by the clinical need for additional methods to investigate factors that may influence agreement among all EER diagnostic tools. Patient compliance may very well be a silent but a salient factor. Although participants in the current study reported high compliance in all test areas, three of the questions having to do with “forgetting” did not show acceptable levels of internal consistency. Exploring alternative ways to estimate patient compliance during pH probe monitoring is an area needing development. It is also unknown how factors such as education, language, psychological characteristics, or socioeconomic status may have affected compliance in the current study. Despite these limitations, a compliance questionnaire can easily be given to a patient following pH testing and should provide additional insight while improving the confidence level of study interpretation. Results highlight the need for further development to strengthen the sensitivity and reliability of the questions.

## 5. Conclusions

This is the first study to investigate participant compliance and accuracy with a standard pH probe test protocol. For all parameters queried, participants reported adherence to typical routines during the 24-hour pH probe test. Furthermore, they reported a high level of compliance to test instructions (reporting ingestions, body position, and symptoms). Efforts to assess patient compliance may increase the confidence with which results may be interpreted, thereby increasing the clinical utility 24-hour dual pH probe testing for EER.

## Figures and Tables

**Table 1:** Means and standard deviations of responses on Post-Evaluation Questionnaire items 1–7.

Variable	Mean	SD
Q1: overall level of discomfort	2.11	1.16
Q2: typical daily activities	1.55	1.63
Q3: typical diet	1.31	1.58
Q4: forgot to change setting for body position	0.45	0.77
Q5: forgot to change setting for eating/drinking	0.55	0.57
Q6: forgot to use symptom button	0.78	1.05
Q7: typical symptoms over past 24 hours	1.64	1.56

## Appendices

### A. pH Probe Food Diary

Please list everything that you ate or drank in the past 24 hours. Please also include the time ranges (start and end times) that you ate/drank (i.e., 11:10–11:34 am).

Breakfast

Food:   
Liquid:
Other (Condiments, Spices, etc.):

Snack: 

Food:   
Liquid:
Other (Condiments, Spices, etc.):

Lunch: 

Food:   
Liquid:
Other (Condiments, Spices, etc.):

Snack: 

Food: 
Liquid:
Other (Condiments, Spices, etc.):

Dinner: 

Food:
Liquid:
Other (Condiments, Spices, etc.):

Snack:

Food:
Liquid:
Other (Condiments, Spices, etc.):

### B. Post-Evaluation Questionnaire

Based on this experience with the pH probe test, please circle the appropriate response.

(1) Please rate your overall level of discomfort during the past 24 hours. (0 = No discomfort, 5 = Severe discomfort)
  0 1 2 3 4 5
(2) How typical were your daily activities during the past 24 hours? (0 = Very typical, 5 = Very atypical/Very different)
  0 1 2 3 4 5
(3) How typical was your diet during the past 24 hours? (0 = Very typical, 5 = Very atypical/Very different)
  0 1 2 3 4 5
(4) How many times did you accidentally forget to change the setting for your body position (upright/lying down) over the past 24 hours? (0 = Never, 1 = One or two times, 2 = Three or four times, 3 = Five or six times, 4 = Seven or eight times, 5 = Nine or more times)
  0 1 2 3 4 5
(5) How many times did you accidentally forget to change the setting for an eating/drinking event over the past 24 hours? (0 = Never, 1 = One or two times, 2 = Three or four times, 3 = Five or six times, 4 = Seven or eight times, 5 = Nine or more times)
  0 1 2 3 4 5
(6) How many times did you accidentally forget to use the symptom button over the past 24 hours? (0 = Never, 1 = One or two times, 2 = Three or four times, 3 = Five or six times, 4 = Seven or eight times, 5 = Nine or more times, 9 = N/A)
  0 1 2 3 4 5 9
(7) How typical were your symptoms of acid reflux over the past 24 hours? (0 = Very typical, 5 = Very atypical/Very different, 9 = N/A)
  0 1 2 3 4 5 9
(8) Did you have foods or beverages that you know aggravate your symptoms of acid reflux over the past 24 hours?
(Please circle one): Yes/Unsure/No/Does not apply
If “Yes” please list items:
